# Changes in resource perception throughout the foraging visit contribute to task specialization in the honey bee *Apis mellifera*

**DOI:** 10.1038/s41598-023-35163-y

**Published:** 2023-05-19

**Authors:** Emilia Moreno, Andrés Arenas

**Affiliations:** 1 Laboratorio de Insectos Sociales, Departamento de Biodiversidad y Biología Experimental, Facultad de Ciencias Exactas y Naturales, Universidad de Buenos Aires, Buenos Aires, Argentina; 2 Instituto de Fisiología, Biología Molecular y Neurociencias (IFIBYNE), CONICET – Universidad de Buenos Aires, Buenos Aires, Argentina

**Keywords:** Zoology, Animal behaviour, Animal physiology, Entomology, Neuroscience, Learning and memory, Olfactory system, Social behaviour

## Abstract

Division of labor is central to the ecological success of social insects. Among foragers of the honey bee, specialization for collecting nectar or pollen correlates with their sensitivity to sucrose. So far, differences in gustatory perception have been mostly studied in bees returning to the hive, but not during foraging. Here, we showed that the phase of the foraging visit (i.e. beginning or end) interacts with foraging specialization (i.e. predisposition to collect pollen or nectar) to modulate sucrose and pollen sensitivity in foragers. In concordance with previous studies, pollen foragers presented higher sucrose responsiveness than nectar foragers at the end of the foraging visit. On the contrary, pollen foragers were less responsive than nectar foragers at the beginning of the visit. Consistently, free-flying foragers accepted less concentrated sucrose solution during pollen gathering than immediately after entering the hive. Pollen perception also changes throughout foraging, as pollen foragers captured at the beginning of the visit learned and retained memories better when they were conditioned with pollen + sucrose as reward than when we used sucrose alone. Altogether, our results support the idea that changes in foragers' perception throughout the foraging visit contributes to task specialization.

## Introduction

Division of labor is a key feature of social insects based on the performance of groups of specialized individuals that carry out several activities simultaneously, enabling colonies to function efficiently. This phenomenon is explained by the response threshold model^1^, which states that individuals differ in their sensitivity (and therefore in their responsiveness) to biologically relevant stimuli associated with specific tasks, leading to the emergence of division of labor^2,3^ Thus, differential responsiveness, or sensitivity, to stimuli that act as a positive (e.g. food) or a negative (e.g. noxious events) reinforcement affects individual learning performances^4–6^.

Pollen (protein supply) and nectar (carbohydrates supply) are the main stimuli that motivates the foraging behavior of the honey bee *Apis mellifera*^7^, and both act as reinforcements during the learning process^8^. Even though both resources are generally available as reward in flowering plants at the same time^9^, honey bee foragers specialize in collecting either pollen or nectar. Foraging specialization is linked to differences in sucrose sensitivity and it is probably the best studied case that assesses variations in behavioral responsiveness in the context of division of labour^10^. However, at first glance, findings on honey bee behavior are not consistent with the hypothesis stated in the response threshold model, as nectar foragers are less sensitive to sucrose (i.e. a major compound of nectar) than pollen foragers. By testing bees captured arriving at the hive entrance, it has been shown that pollen foragers are more responsive to a broad spectrum of sucrose concentrations than nectar foragers, which mainly respond to highly concentrated sucrose solutions. Such perceptual difference has been interpreted as adaptive, as low sucrose sensitivity among nectar foragers may bias searching for productive sources that provide the colony with a higher energy gain^11,12^. Nevertheless, differences in sucrose responsiveness do not fully explain why foragers that are highly sensitive to sucrose are prone to collect pollen.

Pollen foragers learn faster and retain memories better than nectar foragers when pollen is used as reward^13^ and when odors are presented at low intensities^14^. High gustatory and olfactory sensitivity might enable pollen foragers to better assess pollen, which enhances learning of environmental cues and foraging efficiency^14^. It was hypothesized that sucrose sensitivity correlates with behavioral responses triggered by other stimuli such as odors^14^, light^15^, and tastes (e.g. those available in pollen; see ref.^16^. Thus, a high sensitivity of pollen foragers to a wide range of foraging stimuli could explain why they are more sensitive than nectar foragers to sucrose^17^. However, sucrose responsiveness does not always correlate with the behavioral responses (i.e. sensitivity to an electric shock in ref.^18^), indicating that sucrose perception does not account for responsiveness to stimuli in every sensory modality or behavioral context.

Sucrose responsiveness has a genetic basis, and it is affected by the environmental conditions and by the internal state of the bee. Sensitivity to sucrose varies with age, caste, sex, foraging experience, feeding status^19,20^, season^21^, stress (handling), hormone levels, and exposure to pheromones^22–24^. Moreover, taste perception must be strongly influenced by the bee’s motivation to find the wanted resource. It has recently been reported that honey bee appetitive motivation varies along the different phases of the foraging cycle^25^, which is evinced through significant changes in dopaminergic brain levels of nectar foragers. Dopamine levels are high in nectar foragers departing from the hive and arriving at a profitable sugar source, but decrease upon consummation of the goal (i.e. when bees have consumed sucrose solution and returned to the hive). If we consider that motivation for searching the preferred resource changes between arrival at the food source and departure (after foraging), then foragers that arrived at a pollen source would be less responsive to sucrose than nectar foragers. Moreover, the pollen reward would induce better learning performances and less extinction in pollen foragers compared with nectar foragers. Conversely, nectar foragers would show a higher sucrose responsiveness when they arrive at the food source than when returning to the nest. Remaining responsive to a broad spectrum of sugar solutions would be adaptive for bees having the role of collecting nectar.

Gustatory perception studied in foragers captured at the hive entrance is consistent with the hypothesis that, at departure from the food source and after having reached their respective foraging goal, responsiveness increases for pollen foragers to compensate for energetic needs of the flight, while it would decrease for nectar foragers, no longer attracted by a reward that they possess in their crop and which could be used for energetic demands, if any.

Besides previously reported differences between nectar and pollen foragers captured at the hive entrance, differences in responsiveness to sucrose between pollen and nectar foragers arriving at the food source remain to be tested. Here, we hypothesize that foragers' sucrose perception is not the same throughout the foraging visit neither at different stages of the foraging cycle, as it might be influenced by the interactions among internal and external factors such as motivational state, genetic predisposition, sugar satiety level, and perception of contextual cues from the food source and from the nest. We expect that changes in sucrose responsiveness translate into changes in learning and memory extinction.

We showed that the phase of the foraging visit (i.e. arrival or departure from a food source) interacts with foraging specialization (i.e. predisposition to collect pollen or nectar) to modulate sucrose and pollen sensitivity in foragers. In concordance with previous studies, pollen foragers presented higher sucrose responsiveness (lower response thresholds) than nectar foragers at the end of the visit. On the contrary, consistently with the response threshold model and the motivational state of the foraging bees, we observed that pollen foragers were less responsive than nectar foragers when they arrived at the food source. Furthermore, context drastically influenced sucrose mediated response as we showed that free-flying bees were less likely to accept sucrose solution when they were collecting pollen in a feeder than when they return (loaded with pollen) to the hive. Besides sucrose, pollen perception also changes throughout the foraging visit. During an olfactory conditioning, pollen foragers captured when they arrived at the source learned better when we used pollen + sucrose as reward than when we used sucrose alone. Extinction of memories was higher when they were established using sucrose alone than pollen + sucrose as reward during the conditioning. Such differences in memory acquisition and extinction were not detected among departing bees, indicating that pollen contribution as a reward is more important for pollen foragers at the beginning than at the end of the foraging visit. Altogether, our results support the idea that resource perception changes throughout the foraging visit contribute to foraging specialization.

## Results

### Changes in sucrose responsiveness are influenced by foraging specialization and by the phase of the foraging cycle

**Figure 1:**
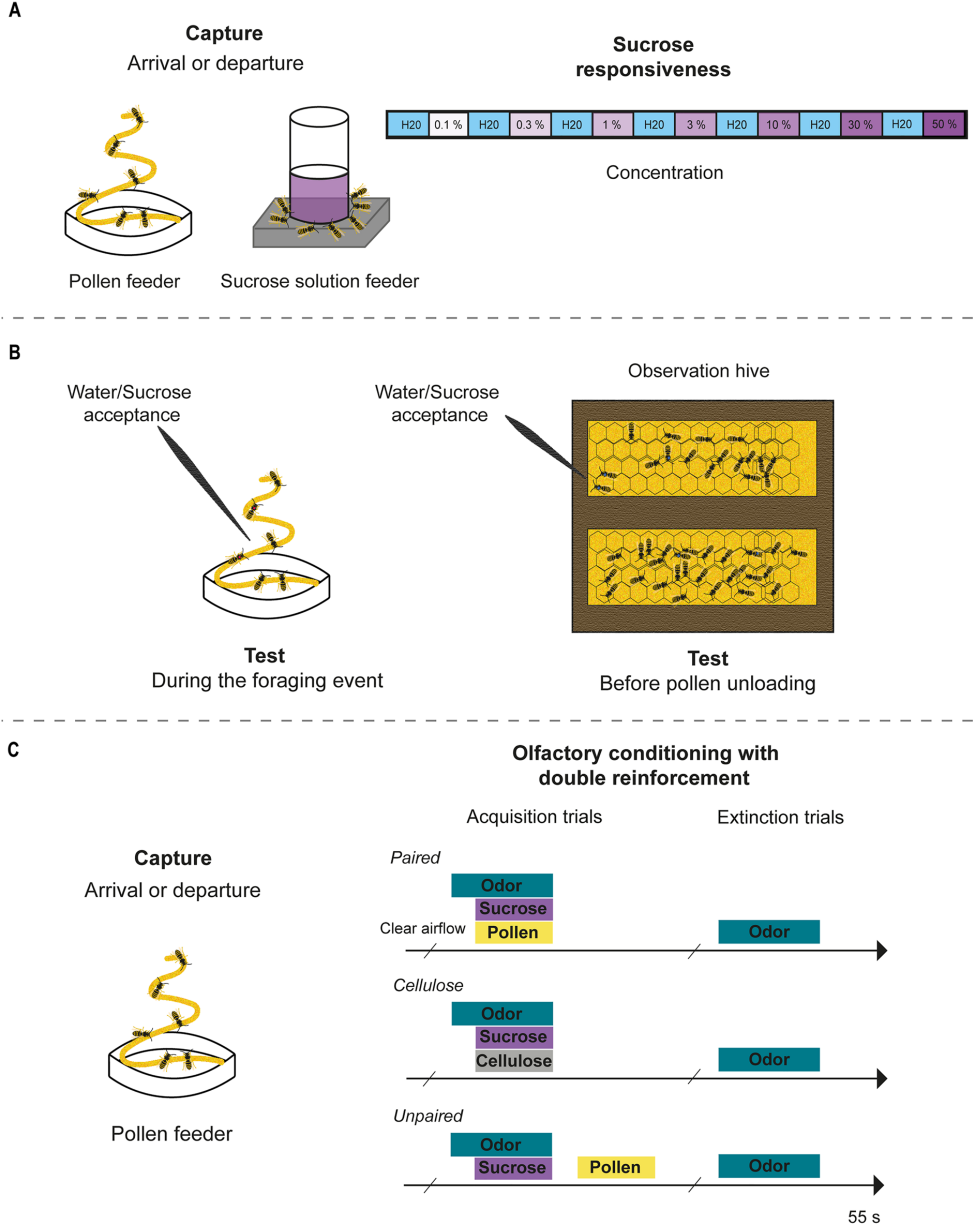
Schematic schedule of the experiments. (**A**) Experiment 1: Sucrose responsiveness. *Ad libitum* nectar and pollen feeders were located on a foraging station 50 m away from the apiary. We captured bees either when they arrived at or departed from the respective *ad libitum* feeder. We harnessed the bees and stimulated their antennae with a series of sucrose solutions of increasing concentrations (0.1, 0.3, 1, 3, 10, 30 and 50%) to determine which solution elicited the extension of the proboscis (PER). Bees were tested in sequential order, starting from de lowest to the highest concentration of sucrose-water solution. We provide bees with water between sucrose trials to prevent responses to sucrose solution caused by thirst. We waited an inter-trial interval of 2 min between each presentation. (**B**) Experiment 2: Sucrose acceptance. We trained a group of bees to collect pure crushed multifloral bee-collected pollen from an artificial *ad libitum* feeder and marked them with acrylic paint of different colors for identification during successive foraging visits. We tested bees' sucrose perception in situ while they were collecting pollen from a feeder or when they entered the hive loaded with pollen corbicules after recollection and before pollen unloading. We contacted the antennae of individual bees with a long stick (15 cm) embedded in sucrose sn. 40% or water. If bees extended their proboscis, we allowed them to ingest a drop of the solution (ca. 7 µl). (**C**) Experiment 3: Olfactory conditioning with co-reinforcement. We captured bees either when they arrived at or when they departed from the* ad libitum* pollen feeder. We harnessed the bees. We olfactory conditioned bees by the presentation of the floral odor linalool (0.1 M, Sigma-Aldrich) as conditioned stimuli to both antennae, sucrose solution (15%) as reward to the antennae and hand-collected kiwi pollen as co-reinforcement to the left first tarsi. Memories formed during 4 acquisition trials were then evaluated along 4 extinction trials that consisted in the presentation of the odor alone.

**Figure 2:**
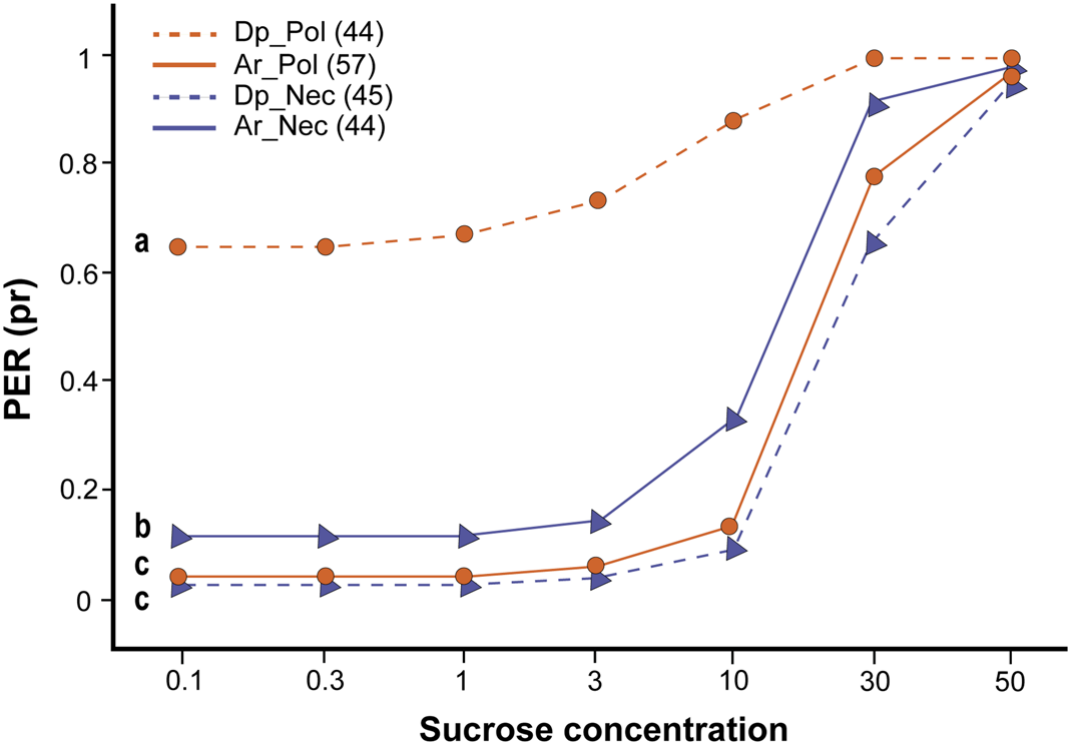
Proportion of PER (pr) of pollen (orange lines) and nectar (blue lines) foragers in response to increasing concentrations of sucrose solution. Dotted lines correspond to departing pollen (Dp_Pol) and nectar (Dp_Nec) foragers, while solid lines correspond to arriving pollen (Ar_pol) and nectar (Ar_Nec) foragers. Different letters indicate statistical differences (p < 0.05). Sample sizes are indicated in parenthesis. We used a GLMM for statistical analysis.

To evaluate sucrose responsiveness, we captured foragers arriving or departing from an artificial feeder with either sucrose solution or crushed bee-collected multifloral pollen (Fig. 1A). Our analysis showed that the forager type interacts with the phase of the foraging visit to affect sucrose responsiveness (χ^2^ = 75.89, df = 1; p < 0.001). Pollen foragers presented higher sucrose responsiveness (lower response thresholds) than nectar foragers at the end of the visit, as we detected significantly higher PER proportions throughout the entire range of evaluated sucrose concentrations (Fig. 2). On the contrary, and consistent with the prediction of the response threshold model, we observed that pollen foragers were less responsive than nectar foragers when they arrived at the foraging station (Fig. 2). Within forager types, pollen foragers were more sensitive to sucrose at the end of the visit (z ratio = – 7.830, p < 0.0001), whereas nectar foragers showed higher sucrose responsiveness at the beginning (z ratio = 3.935, p < 0.0005).

### Changes in sucrose acceptance of free-flying pollen foragers are influenced by the phase of the foraging cycle

**Figure 3:**
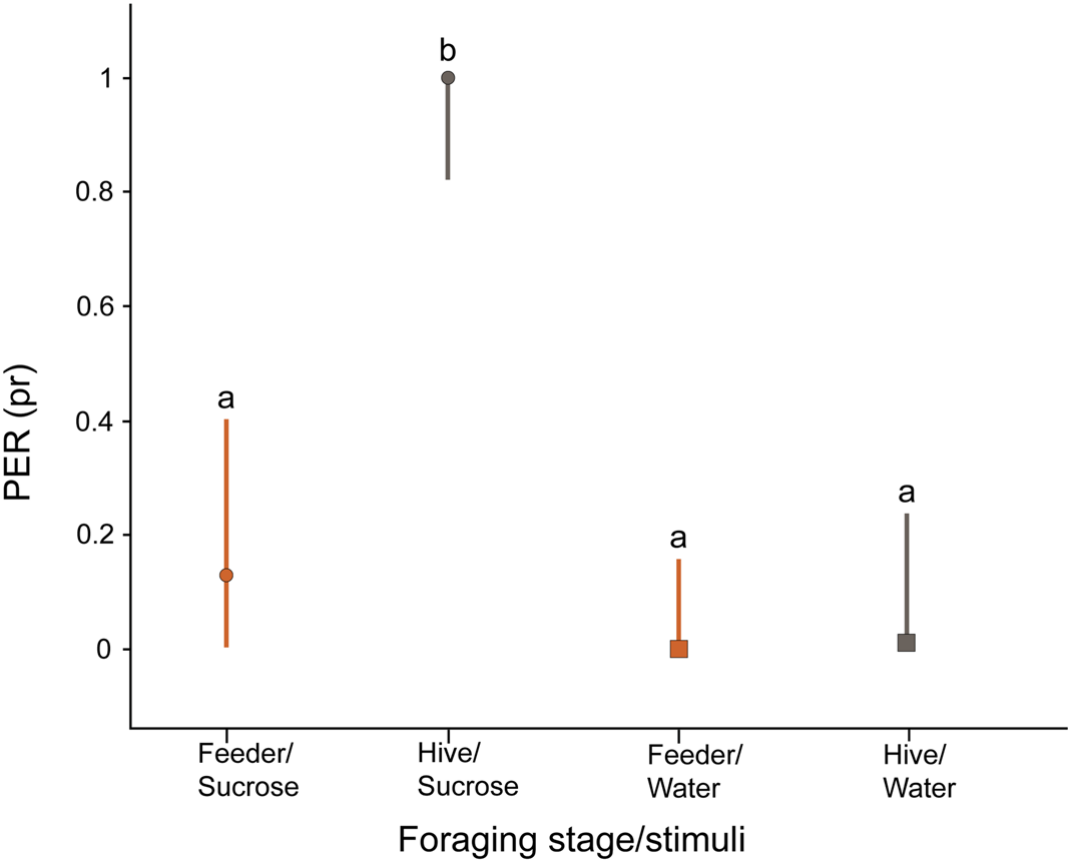
Proportion of PER (pr) of pollen foragers in response to sucrose (circles) or water (squares) during pollen recollection at the feeder (orange lines) or inside the hive (gray lines). Different letters indicate statistical differences (p < 0.05). Sample sizes are FS = 19, HS = 20, FW = 9, HW = 8. We used a GLMM for statistical analysis.

To estimate sucrose acceptance at different stages of the foraging cycle, we tested free-flying bees while they were gathering pollen in an artificial feeder or when they had returned to the hive (loaded with pollen after recollection; Fig. 1B). At the pollen feeder, we observed that only a small proportion of pollen foragers (0.14) accepted a 40% (w/w) sucrose solution, while almost all pollen foragers evaluated immediately after entering the hive accepted it (0.98; Fig. 3). Interestingly, at the feeder, the proportion of bees that responded to sucrose solution did not differ from the proportion of bees that responded to water (z ratio = 2.544 p = 0.534). On the contrary, the differences between sucrose and water acceptance were highly significant inside the hive (z ratio = 3.751, p = 0.001).

### Changes in learning and memory with pollen as reward are influenced by the phase of the foraging cycle

**Figure 4:**
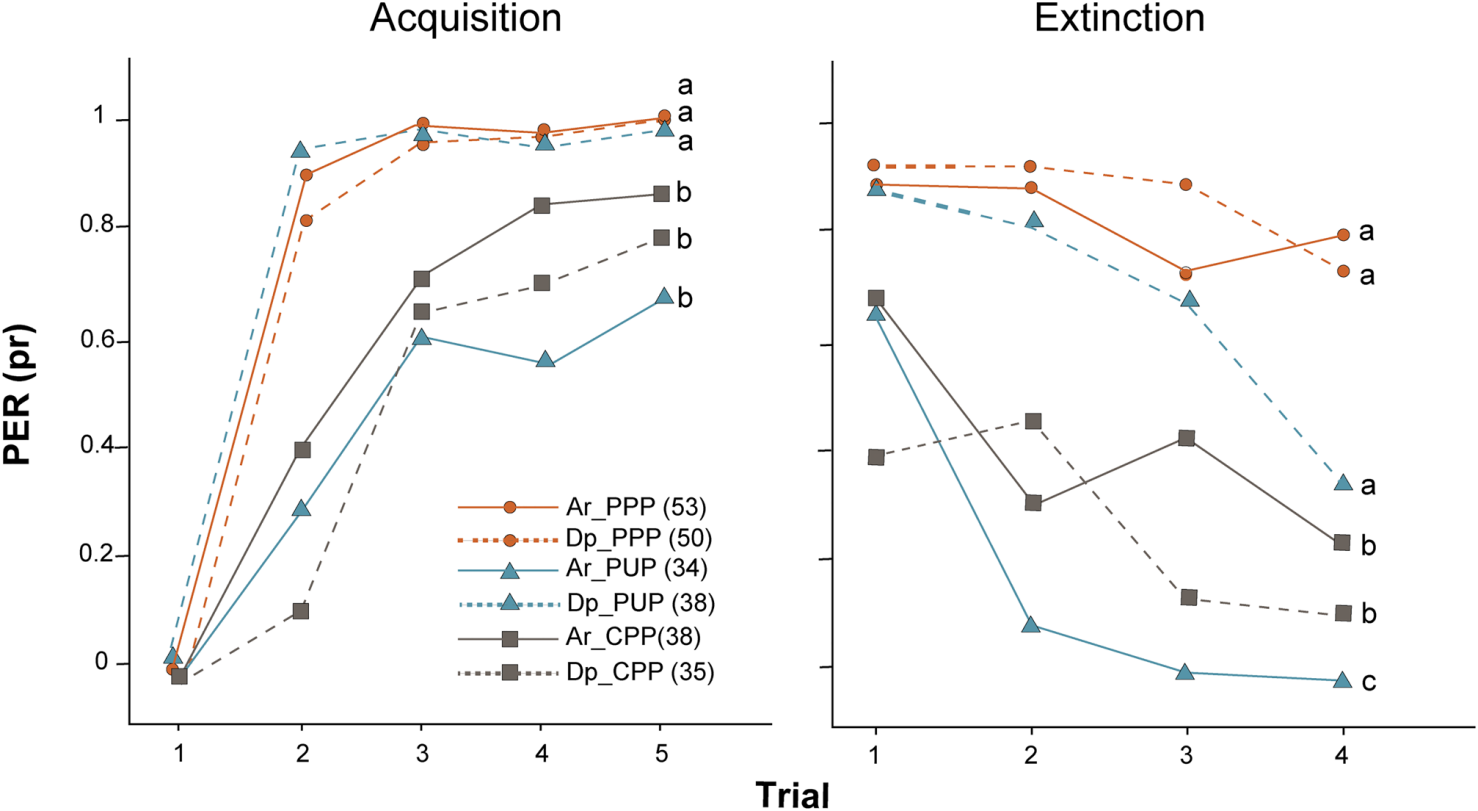
Proportion of PER (pr) responses of pollen foragers during 5 acquisition (left) and 4 extinction (right) trials when pollen foragers arrive (solid lines) or depart (dotted lines) from the feeder. Pollen was used as reinforcement paired (PPP; orange line with circles) or unpaired (PUP; blue line with triangles) with sucrose-water solution. Cellulose (CPP; gray line with squares) was used instead of pollen paired with sucrose-water solution. Different letters indicate statistical differences (p < 0.05). Sample sizes are indicated in parenthesis. We used a GLMM for statistical analysis.

To evaluate how pollen perception changes as a function of the phase of the foraging visit we conditioned pollen foragers to learn olfactory memories using pollen + sucrose as rewards. For this, we subjected foragers that arrived at or departed from a pollen feeder to an olfactory conditioning of PER. After the conditioning procedure, we tested the extinction of olfactory memories by the presentation of the odor alone (Fig. 1C). Our analysis for memory acquisition showed that the PER proportion was influenced by both the moment of the foraging visit and the reinforcement type (χ^2^ = 17.4799, df = 2; p = 0.00016; Fig. 4). Using the double reinforcement (pollen paired procedure or PPP), we observed that pollen foragers acquired olfactory memories equally when they arrived and when they departed from the source (z ratio = 0.145, p = 1). However, when the odor was presented paired with sucrose but uncoupled of pollen (pollen unpaired procedure or PUP), we observed that acquisition performance was lower in arrivals than in departures (z ratio = − 4.212, p = 0.004). Acquisition performance of both arriving and departing bees conditioned under the procedure with cellulose (sucrose paired with cellulose or CPP) was not different from that of arriving bees tested under the unpaired pollen procedure (Ar_CPP: z ratio = − 1.630, p = 0.5784; Dp_CPP: z ratio = − 0.357, p = 0.9992).

For memory extinction, our analysis detected a triple interaction between the stage of the foraging visit, the reinforcement type, and the trial (χ^2^ = 7.9720, df = 2; p = 0.018). As for memory acquisition, pollen foragers with the double reinforcement extinguished olfactory memories equally when they arrived than when they departed from the source (z ratio = − 0.733, p = 0.977). Extinction performance under the unpaired procedure was lower when bees arrived than when they departed from the feeders (z ratio = − 5.002, p =  < 0.001). At arrivals, memory extinction of bees conditioned under the pollen paired procedure was higher than that of bees conditioned under the pollen unpaired (z ratio = − 5.9, p < 0.001) and the cellulose paired procedures (z ratio = − 3.123, p = 0.0221). Our results indicate that pollen contribution as a reward in memory acquisition and extinction is more important when bees arrive at the source (highly motivated) than when they depart from it.

## Discussion

Previous studies demonstrate the relationships between responsiveness of worker honey bees to sucrose and their foraging behavior^11,12^. Here, we show that the timing of the foraging visit is important to the division of labor in the honey bee, as the perception of sucrose that affects foraging specialization increases or decreases throughout the foraging visit according to the motivation for the appropriate reward sought by the individual bees. Similarly, we found that sucrose acceptance varied greatly according to the phase of the foraging cycle, highlighting the idea that reward perception is not fixed but rather rapidly modulated by the context faced by the bee. Moreover, the evidence indicates that the sucrose and pollen sensitivity of pollen foragers varies in opposite directions throughout the foraging visit.

The response threshold model has been extremely influential to explain division of labor and its relation to organization of social insect colonies (e.g.^26,27^). However, the response threshold model itself may be insufficient to account for the division of labor without considerations of the motivational state of the individual and/or its satiety level. From the evidence presented here, motivation seems to be a factor relevant to determine the responsiveness of individuals involved in different tasks.

Gustatory responsiveness remains as the best-studied case of how variations in behavioral responsiveness can result in task specialization. In honey bees, the majority of investigations established differences in the sucrose responsiveness between nectar and pollen foragers that returned to the nest after collecting, but only a few studies analyzed the responses of foragers that departed from the hive, presumably to initiate a foraging trip^11,12^. Using foragers that departed from the hive which were derived from artificially selected high- and low-pollen-hoarding strains, Page et al.^11^ found that bees from the high-pollen-hoarding strain (estimated to be composed of 40% pollen and 60% non-pollen foragers) presented higher sucrose responsiveness than bees from the low-pollen-strain (96% were non-pollen foragers). Although this experiment did not directly evaluate pollen and nectar foragers, it allows us to compare responsiveness of foragers incoming and exiting the hive. Page and coworkers showed that sucrose responsiveness of incoming "nectar foragers” decreased about 25% compared to exiting “nectar foragers”, but that incoming pollen foragers increased by 47% compared to exiting pollen foragers. This evidence is consistent with our results and supports the idea that there is an interplay between the stage of the foraging cycle and foraging predisposition, as the responses of the two groups varied in opposite ways. In another study, Pankiw et al.^12^ tested the sucrose responsiveness in foragers leaving the hive searching for pollen and returning to the hive loaded with pollen. They found that pollen collection did not affect the PER; however, the responsiveness of foragers that arrived at the hive was lower than those foragers exiting the nest.

Changes in perception throughout the foraging visit would not be restricted to gustatory stimuli, and might involve other related stimuli that assist foraging, such as odors. Previous studies have found differences in sensitivity to floral odors between pollen and nectar foragers, with the former group being better at detecting and learning odors presented at low concentrations than nectar foragers (e.g. odors emitted by pollen; see ref.^14^). Such differences were measured in nectar foragers captured while foraging at a sucrose solution feeder (i.e. near departure) while pollen foragers were captured with pollen loads at the entrance of the hive, meaning that both groups were close to the end of their foraging visit. Further experiments are needed to determine whether differences in olfactory sensitivity are also present between foragers arriving at the food sources.

At the food source, perception of innate cues, such as pollen odors, but also learned cues (e.g. odors, colors or shapes) might influence reward perception. Considering that in our experiment bees had previous experiences with the sources (i.e. feeders were available for several days prior to measurements) memories might have affected gustatory perception^28^. Evoking memories established with pollen or sugar might reduce the uncertainty about the reward offered at the foraging site, allowing bees to adjust their sensitivity to their specific expectations, further than bees that have no previous experience at the food source. While the presentation of learned odors associated with nectar sensitizes bees by increasing the probability to extend the proboscis^29^, a response devoted primarily to the ingestion of liquid foods, stimuli learned with pollen could instead inhibit the proboscis extension. This idea is supported by the fact that learned odors at pollen sources bias e.g. the orientation of foragers in a Y-maze, but do not induce the extension of the proboscis upon the odor presentation^30^. Further experiments comparing the changes in the gustatory responsiveness of single foragers through different stages of the foraging cycle (including arriving and departing from the food source, during recollection, and incoming and exiting the hive) will allow us to better understand reward perception during foraging.

Based on our results, we can assume that responsiveness to sucrose changes rapidly between arrival and departure from a food source (events elapsed between a few seconds and a few minutes). Such changes can be explained by rapid variations in the brain levels of biogenic amines (or their receptors) that affect responsiveness linked to different motivational states^25,31^. However, the procedure used to measure sucrose responsiveness is time consuming and the delay between capture and measurement might interfere with the motivational state of the bee. In addition, we cannot rule out possible changes in perception related to the stress of being harnessed in the tubes. On the contrary, and although the information it provides does not allow estimating sucrose sensitivity, sucrose acceptance enables establishing the proportion of free-flying bees responding to a given sucrose solution, at the precise point when the forager (i) starts collecting pollen or (ii) enters the nest with pollen loads (exp. 2; Fig. 3). Interestingly, sucrose acceptance in foragers gathering pollen was extremely low (0.18) compared to pollen foragers tested for similar concentrated solutions in a restrained situation (0.66). Such difference is consistent with a stress-reduced assessment and with the idea that the cues that surround the sources affect the resource perception. In this regard, we know that pollen volatiles, such as those available in exp. 2, are useful to contextualize the foraging site, as these odors are used by bees as indicators of source productivity^32^.

A recent experiment, in which gene expression of octopamine receptors were quantified in foragers captured immediately after landing at feeders, showed that there was an overall higher expression of the receptor gene AmoctaR1 in the brain of pollen foragers compared to nectar foragers^31^. Interestingly, pollen and nectar foragers captured at the end of their foraging bout did not differ in AmoctαR1 expression, but they do show differences for Amtyr1 in the subesophagic zone. A qualitative comparison between pollen foragers captured when arriving at the feeder and captured at the entrance of the hive suggests that expression levels of AmoctαR1 gene is high at the beginning of the foraging trip but downregulated as the foragers become loaded. Once inside the hive, this receptor might be upregulated again, driving the bees to resume pollen foraging. In that sense, a recent work in honey bee nectar foragers evidenced motivational variation according to the phase of the foraging cycle^25^. This work showed that dopamine levels are higher in foragers departing from the hive and arriving at a profitable sugar source but decrease upon consummation of the goal (i.e. when bees have consumed sucrose solution and return to the hive). Thus, it is likely that specific biogenic amine profiles, expression levels of their receptors, or even variations of other molecular actors that mediate appetitive responses (e.g. small neuropeptide F; ref.^33^) are responsible for driving pollen and nectar foragers throughout the different phases of the foraging bout^31^.

There are fundamental differences in how bees collect and transport nectar and pollen. Foraging trips involve visiting hundreds of flowers to collect a few microliters of nectar or a few micrograms of pollen before returning to the hive to unload, and then depart again for another foraging trip^7^. Once nectar is ingested, it is stored in a portion of the digestive tract (the crop), where it is transported to the hive. On the contrary, pollen is agglutinated during recollection and transported in specialized structures on the hind legs, the corbiculae^34^. In addition, pollen foragers use nectar to aggregate pollen grains into their corbiculae. Inside the hive, foragers consume honey as fuel before exiting the nest, so technically they do not need to ingest food during foraging activity. However, bees can use stored nectar from the crop to provide an immediate energy supply if they face high energy demands^35^. Because unlike other insect groups, honey bees cannot use protein constituents such as proline to fuel their flight muscles^36^, pollen foraging activity might be highly restricted to the initial amount of nectar carried in their crop (i.e. nectar received from nest mates is the lonely fuel pollen foragers had to fulfill their flights), limiting the duration of the visit and the amount of pollen that bees can collect. Thus, pollen and nectar foragers may greatly differ in their sugar satiety level along the foraging cycle, with pollen collection generating substantially higher sugar demands. In this regard, Harano and Nakamura^37^ examined the concentration of nectar carried from the nest according to the type of foragers and their need for sugar. In line with our results, they found that pollen foragers had more concentrated nectar (61.8% w/w) than nectar foragers (43.8% w/w) as their energetic demands are greater, suggesting that the sucrose response threshold of pollen foragers leaving the nest is higher than those of nectar foragers.

Because sucrose responsiveness varies due to the bee´s nutritional status, both pollen and nectar foragers significantly reduce their sensitivity to sucrose if satiated^11,12^. However, differences in sucrose sensitivity between forager types remain detectable after feeding, suggesting that genetic-based foraging predisposition accounts for differential responses^11^. Other than genetic bases, exposure to local signals, such as pheromones, or experiences acquired while the bee specializes in collecting either pollen or nectar, might contribute to those differential responses too. It remains an open question whether the same results for sucrose responsiveness will be obtained if we use more distant food sources. Average foraging distances are usually longer than those we used in our experiments, in which we trained bees to visit feeders located 50 m away from the hives, a condition that we might consider to be realistic (e.g. bees can collect resources at very short distances^38,39^). Foragers adjust the amount and concentration of the nectar they carry in their crop according to the distance to the source^37^. Thus, we expect pollen foragers returning from a distant source to arrive at their hive with little or no nectar (fuel) in their crop, similar to foragers returning from a nearby source. However, when leaving a distant source, pollen foragers may contain a greater crop load than foragers frequenting nearby sources, which could result in lower levels of sucrose sensitivity. Despite such putative differences, we speculate that response patterns throughout the foraging visit vary similarly within long and short distance foraging bees.

Although most studies focused on sucrose responsiveness, a few experiments have also measured gustatory sensitivity to pollen^11,17^. Page and coworkers^11^ observed that pollen foragers were more likely to show the PER than nectar foragers when they were stimulated using pollen loads. Interestingly, they noticed that such differences in pollen perception became non-significant after feeding the bees with sucrose, suggesting that differences in bees' responses were triggered by sugars of pollen. Using the procedure of co-reinforcement of sucrose and pollen we showed that foragers returning to the hive loaded with pollen performed better during an olfactory conditioning than foragers captured while collecting nectar from an artificial feeder^13^. Our results show that pollen foragers improved their performance when conditioned with pollen and sucrose as reward if captured upon arrival at the food source, but not when captured at departure. This suggests that the contribution of pollen as a reward is higher upon arrival than when bees prepare to leave from the foraging site. It is interesting to note that cellulose presentation also improved the performance of bees arriving at the source (evidenced mainly during memory extinction), suggesting that a tactile stimulation with inert particles similar in size to pollen grains is perceived as an appetitive stimulus too. In contrast to previous findings that showed a positive correlation between sucrose and pollen sensitivity^11,17^, our results indicate that pollen foragers that arrived at the sources are very sensitive to pollen but no to sucrose. Even when bees can extend their proboscis by pollen stimulation^11,17,40^, PER may not be the most appropriate paradigm for assessing responses related to pollen foraging, as this is a response closely associated with ingestion of liquid food. Therefore, we do not rule out the possibility that there are other procedures by which we can further explore pollen sensitivity in honey bees.

## Methods

### Study site

We carried out experiments 1 and 3 during the summer seasons of 2020–2021 and experiment 2 during the summer season of 2019 in the Experimental Field of the Faculty of Exact and Natural Sciences of the University of Buenos Aires (UBA), Argentina. We performed all experiments according to the animal care guidelines of the National Institute of Health (1985) and the current laws of Argentina.

### Experimental bees

We tested European honey bees *A. mellifera ligustica.* Foragers were trained to visit a foraging station located 50 m away from the apiary. We trained the bees using a 10% sucrose solution and crushed bee-collected multifloral pollen, which were offered in separate (20 cm apart) *ad libitum* feeders located on a wooden platform (30 cm x 40 cm).

### Experiment 1 Testing sucrose perception

In this experiment, we assessed sucrose responsiveness of pollen and nectar foragers at different phases of their foraging visit. Sucrose sensitivity can be evaluated by means of the proboscis extension reflex (PER); an innate response triggered after touching the bee's antennae with a sufficiently concentrated sucrose solution^41^. We stimulated the antennae of restrained bees with a series of sucrose-water solutions of increasing concentrations (0.1, 0.3, 1, 3, 10, 30 and 50%) to determine which solution elicited the extension of the proboscis. We captured bees when they arrived (once they landed on the feeder’s surface, before resource recollection) and when they departed (once they cleaned their antennae, after resource recollection). In that way, we obtained 4 different groups of foragers: (i) pollen-arrival, (ii) pollen-departure, (iii) nectar-arrival, and (iv) nectar-departure. In the laboratory, we chilled bees in the freezer until they remained immobile and carefully restrained them in harnesses that only allowed their antennae and mouthparts to move freely^42,43^. We offered water with a toothpick until satiation before we placed them into the incubator (30 °C, 60% RH, and darkness) for 30 to 45 min. We did not feed bees during trials to not modify their motivation to respond. Bees were tested in sequential order, starting from de lowest to the highest concentration of sucrose solution with an inter-trial interval of 2 min (Fig. 1A). We provide bees with water between sucrose trials to prevent responses to sucrose solution caused by thirst^11^.

### Experiment 2 Testing sucrose acceptance

We studied the extent to which manipulation and context (i.e. foraging or social context) affect foragers' acceptance of sucrose (including the ingestion of a small sample of sucrose solution). Here, we tested bees' sucrose perception in situ in individuals that were collecting pollen from a feeder and in bees entering the hive loaded with pollen corbicules after recollection. For the first situation, we trained a group of bees to collect pure crushed multifloral bee-collected pollen from an artificial *ad libitum* feeder and marked them with acrylic paint of different colors to identify them during successive foraging visits. We touched the antennae of individual bees with a long stick (15 cm) embedded in sucrose sn. 40% or water shortly after the bee landed and started manipulating pollen. If bees extended their proboscis, we allowed them to ingest a drop of the solution (ca. 7 µl). The trials lasted 45 min, during which each bee made 3 to 5 trips. To measure acceptance inside the hive, we trained bees obtained from an observation hive to collect pollen from an artificial *ad libitum* feeder containing crushed bee-collected multifloral pollen. As before, we color-marked bees at the feeder to identify them at the foraging station and inside the hive. We measured sucrose and water acceptance immediately after focal bees enter the hive (before they unloaded their corbiculae in the hive cells; Fig. 1B). We removed the sides of the hive, which allowed us to access focal bees easily. Sucrose solution or water was randomly assigned, and each bee was presented with the same solution throughout all visits. When possible, we tested acceptance once per visit throughout all foraging visits or hive stays.

### Experiment 3 Testing learning and memory using pollen as co-reinforcement

We studied the differences in the acquisition and extinction of olfactory memories during associative learning when foragers arrived to and departed from a feeder. As with nectar, bees also extend their proboscis when stimulated with pollen, however; this response is not stable, and bees often stop responding to pollen after a few events. Hence, we developed an alternative procedure in which we offer a simultaneous double reinforcement presenting sugar on the antennae and pollen on the tarsus of the first pair of legs. This procedure allows us to obtain stable PER responses throughout the successive training events. We olfactory conditioned bees by the presentation of the floral odor linalool (0.1 M, Sigma- Aldrich) as conditioned stimulus to both antennae, sucrose-water solution (15%) as reward presented also to both antennae, and hand-collected kiwi pollen as reinforcement to the left tarsus Pollen reward was always presented on the left tarsus to reduce a possible source of variation in the case of lateralization^44^. Memories were formed during 4 acquisition trials followed by 4 extinction trials that consisted in the presentation of the odor alone. We delivered the odor paired with sucrose + pollen (pollen paired procedure, PPP). To control possible sensitization produced by the effect of pollen stimulation on bees, we presented the odor paired with sucrose but not with pollen (pollen unpaired procedure, PUP). To control for mechanical stimulation, we presented the odor paired with sucrose + cellulose (cellulose paired procedure, CPP; Fig. 1C). Despite being an inert compound, cellulose particles (which are similar in size to pollen grains) might provide a tactile stimulation that reinforces learning and memory of bees approaching the pollen source. Finally, bees that showed a spontaneous response (i.e. extending the proboscis in response to the first odor presentation) were excluded, as we cannot determine whether this is an innate response or if it indicates a prior (uncontrolled) odor- reward association as preconized in ref.^45^.

### Odor delivery

To present the CS, we used an olfactometer that sent a continuous clean air flow (50 ml s^–1^) to the head and delivered the odor through a secondary air stream (6.25 ml s^–1^) which was injected into the main airflow through a system of valves controlled by computer. A piece of filter paper (30 × 3 mm) was impregnated with an aliquot of the odor (4μL) and placed inside a syringe connected to the secondary air stream. Each trial lasted 55 s. The valve opening was programmed so that it released clean air during the first 20 s, followed by the odor (6 s), and a final exposure to clean air for the last 29 s. The last 3 s of the odor presentation overlapped with the sucrose + pollen (or cellulose) presentation in the paired procedures. During the unpaired procedures, we presented pollen or cellulose 5 s after the odor + sucrose presentation. We measured the PER during the first 3 s of the odor presentation.

### Statistical analysis

For experiments 1 and 3 we used multiplicative generalized linear mixed models (GLMMs) assuming a Bernoulli distribution. When the PER occurred, we assigned values of 1, and when it did not, we assigned values of 0. In experiment 1, we analyzed the proportion of PER of restrained foragers considering forager type (a two-level factor corresponding to pollen and nectar foragers), foraging stage (a two-level factor corresponding to beginning and end), and sucrose concentration (continuous variable) as fixed effects. Individual bees and experimental days were considered as random effects. For experiment 2, we analyzed the PER proportion in response to sucrose and to water (a two-level factor) when they arrived at the feeder and when they entered to the hive (a two-level factor) by means of a binomial multiplicative generalized linear mixed model, using the “glmmTMB'' function of the ‘glmmTMB’ package^46^. Experimental days were considered as random effects. In experiment 3, we analyzed the proportion of PER of restrained pollen foragers during acquisition and extinction trials considering foraging stage (a two-level factor corresponding to beginning and end), treatment (a three-level factor corresponding to the reinforcement: paired pollen, unpaired pollen and cellulose), and trial (continuous variable) as fixed effects. Each bee was considered as random effects. We used the “glmer” function of the ‘lme4’ package^46,47^. For all the experiments, we conducted post hoc contrasts on models to assess effects and significance between fixed factors using the “emmeans'' function of the ‘emmeans’ package version 1.4^48^ with a significance level of 0.05.

## Supplementary Information

Supplementary Tables.

## Data Availability

All data generated or analyzed during this study are included in this published article [and its Supplementary Information files].
